# A Ghost in Coronary Artery – Coronary Artery Embolism After Discontinuation of Rivaroxaban in a Patient With Atrial Fibrillation: Case Report and Review of Literature

**DOI:** 10.7759/cureus.10082

**Published:** 2020-08-27

**Authors:** Danish Abbasi, Negar Salehi, Saif Faiek, Waqas J Siddiqui, Shahzed Ahmad

**Affiliations:** 1 Cardiovascular Diseases, University of Arkansas, Little Rock, USA; 2 Internal Medicine - Cardiology, University of Arkansas, Little Rock, USA; 3 Internal Medicine, AtlantiCare Regional Medical Center, Atlantic City, USA; 4 Cardiology/Nephrology, Drexel University College of Medicine, Philadelphia, USA; 5 Cardiology/Nephrology, Orange Park Medical Center, Orange Park, USA; 6 Cardiovascular Disease, Lower Bucks Hospital, Bristol, USA

**Keywords:** coronary artery embolism, acute myocardial infarction, rivaroxaban, xarelto, anticoagulation

## Abstract

Coronary artery embolism (CAE) is a rare clinical entity that can cause acute myocardial infarction (AMI). The exact prevalence of coronary artery embolism is unknown. CAE was found to be associated with conditions that can lead to thrombo-embolism, including infective endocarditis, atrial fibrillation, mitral valve disease, valve surgery. Herein, we report a 78-year-old male with a past medical history of atrial fibrillation on rivaroxaban who presented to the hospital emergency department complaining of chest pain. The patient's anticoagulation therapy was recently held due to a concern for gastrointestinal bleeding. After further evaluation of the patient's symptoms and reviewing his electrocardiogram (ECG) which showed ST-depression in lateral leads and ST-elevation in aVR, urgent cardiac catheterization was done which showed left main coronary artery thrombosis extending into the left anterior descending artery (LAD) and left circumflex artery (LCX). The patient was started on a heparin drip and underwent a successful aspiration thrombectomy with subsequent improvement in his symptoms.

## Introduction

Coronary artery embolism (CAE) is a rare cause of acute myocardial infarction (AMI) and should be suspected when AMI occurs in patients with conditions associated with embolism. It is associated with high mortality. Atrial fibrillation (AF) is an associated risk factor for CAE. Discontinuation of anticoagulation, especially rivaroxaban, is associated with a higher risk of embolism [1, 2]. In 1953 Cheng et al. reported 53 cases of CAE. Most of them were diagnosed on autopsy [3]. Before 1960, more than half of CAE were secondary to infective endocarditis [4-6]; however, recent data has shown that coronary embolism is mainly related to atrial fibrillation [6, 7].

## Case presentation

**Figure 1:**
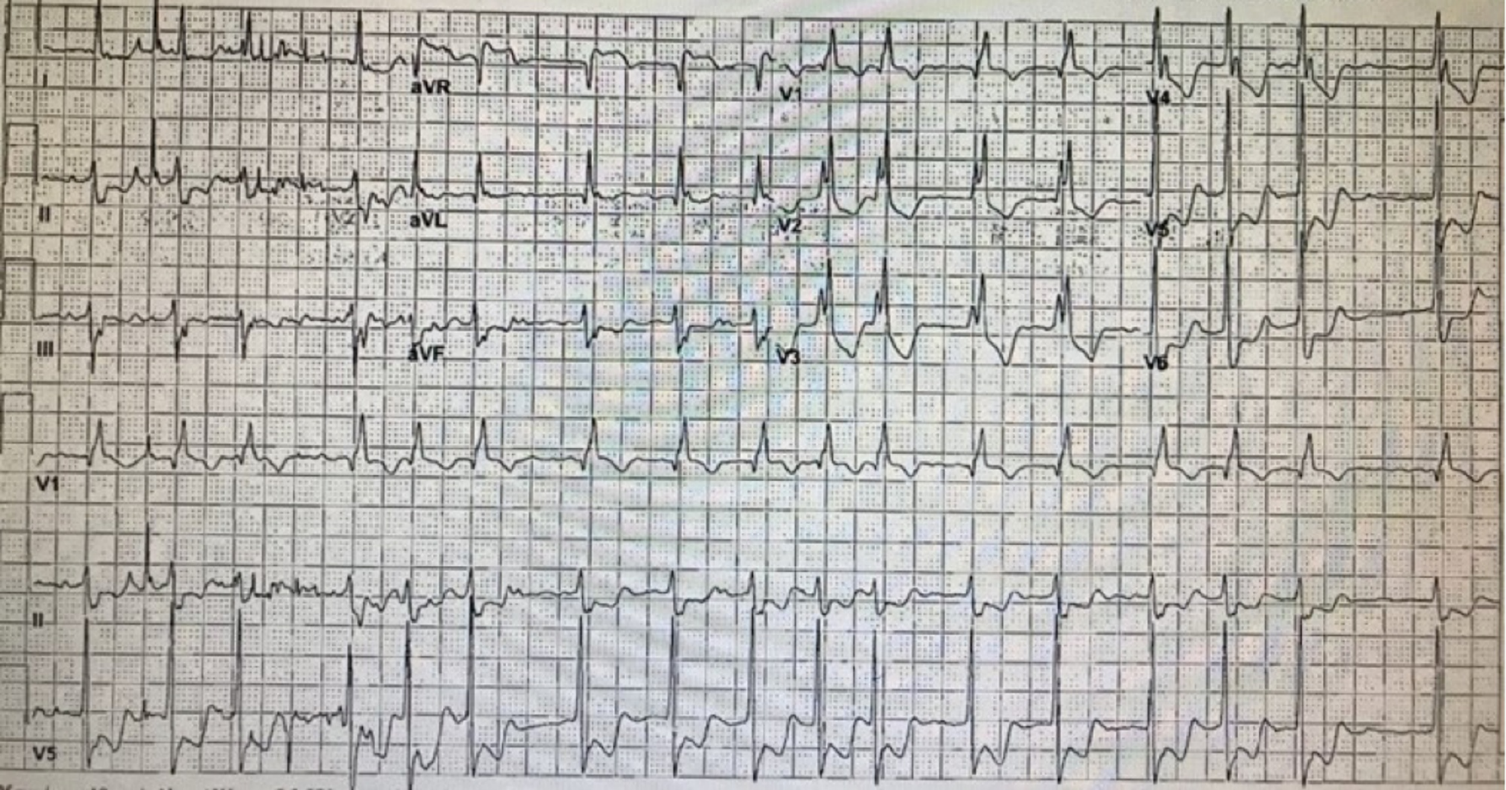
ECG - ST-elevation in aVR, depression in V5 and V6

A 78-year-old male with a past medical history of hypertension, chronic kidney disease, chronic obstructive pulmonary disease, and atrial fibrillation with CHA2DS2-VASc score of 3 on rivaroxaban, presented to the hospital with complaints of left upper quadrant pain and chest pain. The patient was recently admitted with a concern for gastrointestinal bleeding, and rivaroxaban was held. Unfortunately, the patient left the hospital against medical advice at that time without further workup for gastrointestinal bleeding. The patient presented two days after leaving the hospital. On physical examination, the patient was alert and oriented. The abdomen was soft with mild left upper quadrant tenderness. Lungs were clear to auscultation without any crackles or wheezing. The patient had an irregular heart rhythm, and no murmurs were appreciated. The patient's vital signs were stable, except for his heart rate of 100 bpm. The patient underwent a stat computed tomography angiography of the chest, which was negative for aortic dissection and pulmonary embolism. Other laboratory workup showed hemoglobin 9.1 gm/dl (reference range 13-17 gm/dl), hematocrit 27.5% (reference range 41-50%), and lipase 170 U/L (reference range 0-160 U/L). The patient had an electrocardiogram (ECG), which showed atrial fibrillation with a ventricular rate of 112 bpm, right bundle branch block, ST-segment depression in lateral leads with ST-segment elevation in aVR (Figure 1). His Troponin level was minimally elevated at 0.036 ng/ml (normal range below 0.04 ng/ml) with a brain natriuretic peptide (BNP) of 436 pg/ml (normal range below 100 pg/ml).

**Figure 2:**
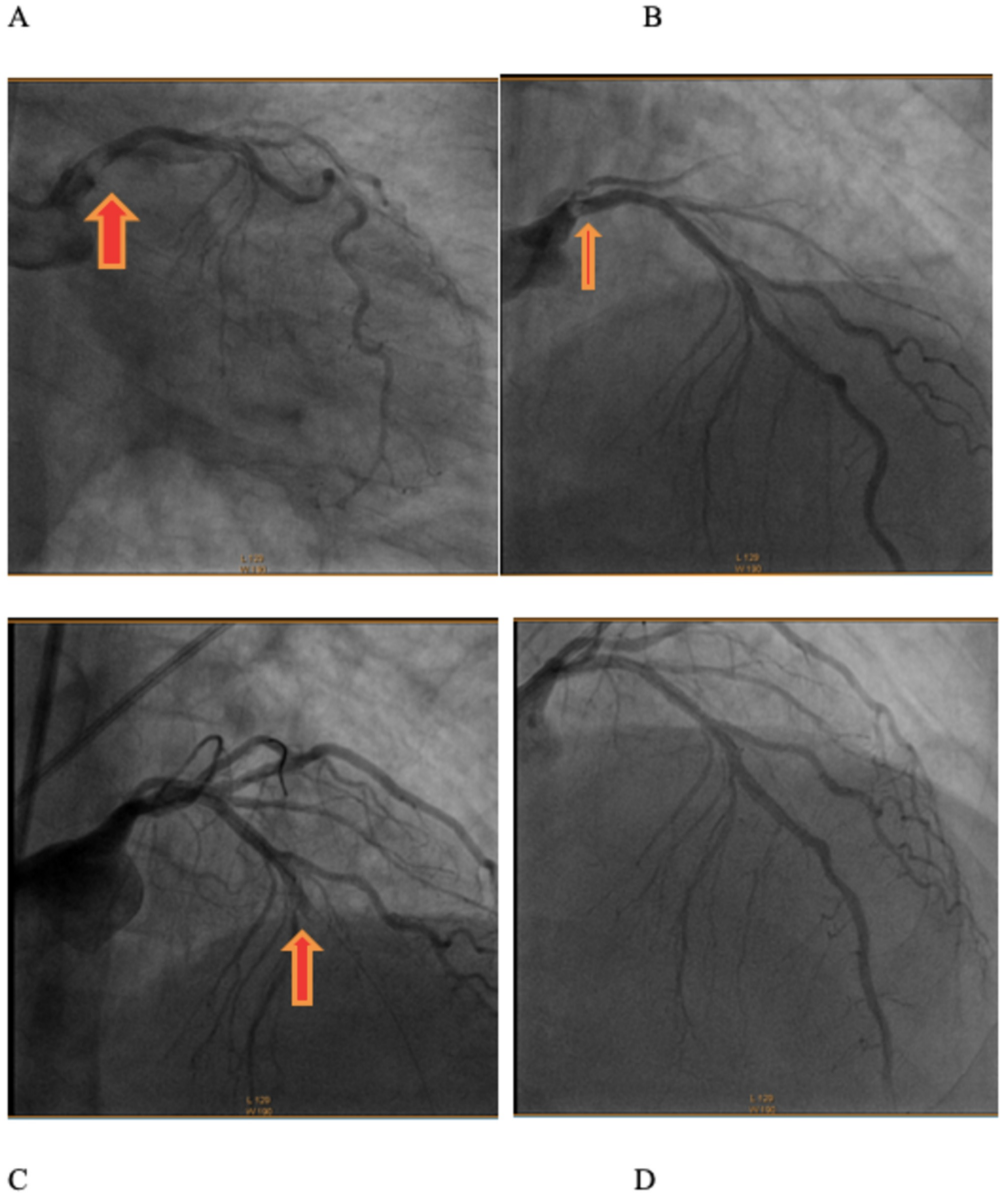
(A) Left coronary angiogram showing left- main thrombus. (B) Left angiogram showing left main, circumflex and ostial LAD thrombus. (C) Left coronary angiogram showing mid LAD occlusion due to distal embolization of thrombus. Note restoration of TIMI grade 3 flow into LCX and clear left-main. (D) Left coronary angiogram showing clear left main and left circumflex artery with distal LAD thrombus. LAD: Left anterior descending artery; LCX: Left circumflex artery.

**Figure 3:**
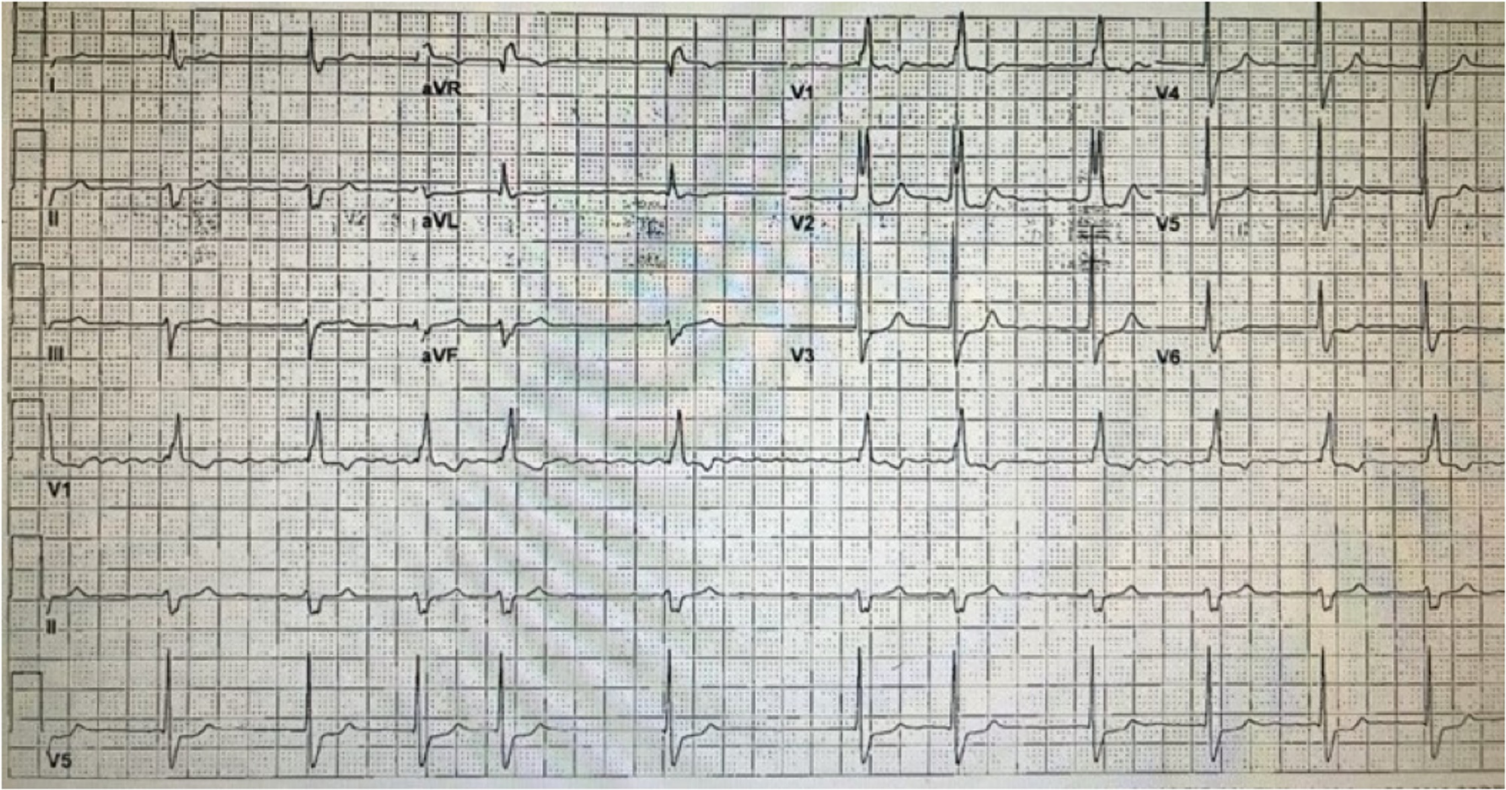
ECG - Resolution of ST-segment changes after intervention

The patient was transferred for urgent cardiac catheterization after heparin drip initiation, which showed left-main thrombosis with extension into the left anterior descending artery (LAD) with 90% obstruction and left circumflex artery (LCX) leading to 100% occlusion without collaterals (Figure 2A, 2B). The mid to distal right coronary artery (RCA) had minimal luminal irregularities. After insertion of the intra-aortic balloon pump (IABP) for hemodynamic support, the left main was engaged, and two 0.014 wires were crossed across the LAD and LCX lesions. Aspiration thrombectomy of LAD was performed that caused the distal embolization of the thrombus (Figure 2C). Repeat thrombectomy was done. Post-procedure there was TIMI grade 3 flow throughout the LCX (Figure 2D) without any evidence of thrombosis, dissection, or distal embolization. The patient transferred to the critical care unit. The heparin infusion and abciximab were continued, repeated ECG showed complete resolution of ST-segment elevation in aVR and depression in anterolateral leads (Figure 3). The patient was subsequently taken off IABP support. The patient was stable without any chest pain and was discharged on rivaroxaban.

## Discussion

Coronary artery embolism is a rare cause of acute myocardial infarction and should be suspected in patients with conditions associated with thrombo-embolism. Virchow described the first case of this type in 1856 [1]. Saphir in 1933 published a review of 16 cases of coronary embolism, including a report of three individual cases [2]. By 1953 Cheng et al. reported 53 cases of CAE. Most of the cases diagnosed on autopsy; only three diagnosed during life [3]. Before 1960, infective endocarditis [4-6] accounted for more than half of all cases; however, the rate has declined since then [1]. Recent data has shown that coronary embolism is mainly related to atrial fibrillation [6, 7]. There are other reports about CAE that included but are not limited to mitral valve disease [8], pulmonary resection [9] and prosthetic heart valve [10].

The exact prevalence of coronary artery embolism is unknown. An extensive review by Shibata et al. [11] of the National Cerebral and Cardiovascular Center (NCVC) AMI database noted that the prevalence of CAE was 2.9% (n = 52), including eight (15%) patients with multi-vessel CAE. The study reviewed 1,776 patients with de novo AMI and found that the most common cause was atrial fibrillation (73%). Five patients of atrial fibrillation had a recurrence CAE with a median follow-up of 49 months, CAE, and thromboembolism recurred in five AF patients. Prizel et al. in 1978 analyzed autopsies of 55 patients with CAE [12]. The study found heart disease (40%), cardiomyopathy (29%), coronary atherosclerosis (16%), and chronic atrial fibrillation (24%) in those patients. Mural thrombi were present in 18 (33%), with clinically diagnosed myocardial infarction in 15 (27%) and death in 11 (20%) patients. Most emboli involved the left coronary artery. The authors of the study concluded that coronary emboli might produce signs and symptoms identical to atherosclerotic coronary disease. Distal embolization was a common cause of infarcts that resulted in small transmural myocardial infarction. Kolodgie et al. noted that coronary arteries are relatively protected from emboli, in comparison with other organs [13]. Reasons for decreased reported incidence also include failure to distinguish embolism from thrombosis or an inability to do a systematic search for small emboli in the distal and intramural branches of coronary arteries. The two significant determinants are the size of the embolus and the size of the lumen of the artery that will be affected. Smaller emboli are more likely to go distal to a small coronary arterial segment and decrease the probability of MI or fatal arrhythmia [14].

Prompt identification and management are essential, as the higher incidence of all-cause death (hazard ratio, 3.82) and cardiac death (hazard ratio, 5.39) have been reported in patients with CAE as compared to non-CAE cohorts. Like in our patient, the DeWinter findings on the EKG of aVR elevation and ST-segment depression in precordial leads were suggestive of left main occlusion or proximal LAD stenosis. Patients with coronary artery embolism appear to be a high-risk sub-group of acute myocardial infarction individuals in the long term. Kaplan-Meier analysis of patients by Shibata et al. also showed similar findings (hazard ratio, 9.29) [11]. The unexpectedly higher mortality rates in the CAE group may be related to comorbidities or lack of collaterals in the native arteries that cause more extensive infarct and more muscle loss.

A coronary angiogram is a diagnostic procedure of choice for CAE. The NCVC proposed three major and three minor criteria for the clinical diagnosis of CAE, which is most commonly used [15]. Diagnosis of coronary embolism is classified as Definite (when two or more major criteria, or one major plus ≥2 minor criteria, or three minor criteria are present) vs. Probable (when one major criterion plus one minor criterion, or two minor criteria are present) (Table 1).

**Table 1:** Proposed National Cerebral and Cardiovascular Center criteria for the clinical diagnosis of coronary artery embolism

Major criteria: 1- Angiographic evidence of coronary artery embolism and thrombosis without atherosclerotic components. 2- Concomitant coronary artery embolization at multiple sites. 3- Concomitant systemic embolization without left ventricular thrombus attributable to acute myocardial infarction.
Minor criteria: 1- <25% stenosis on coronary angiography, except for the culprit lesion. 2- Evidence of an embolic source based on transthoracic echocardiography, transesophageal echocardiography, computed tomography, or MRI (magnetic resonance imaging). 3- Presence of embolic risk factors: Atrial fibrillation, cardiomyopathy, rheumatic valve disease, prosthetic heart valve, patent foramen ovale, atrial septal defect, history of cardiac surgery, infective endocarditis, or hypercoagulable state.
Definite CAE (coronary artery embolism): two or more major criteria, or one major criterion plus ≥2 minor criteria, or three minor criteria
Probable CAE: one major criterion plus one minor criterion, or two minor criteria.
A diagnosis of CE should not be made if there is: pathological evidence of atherosclerotic thrombus, history of coronary revascularization, coronary artery ectasia, plaque disruption or erosion detected by intravascular ultrasound or optic coherence tomography in the proximal part of the culprit lesion

Diagnosis of CAE should not be made if there is pathological evidence of atherosclerotic thrombus, history of coronary revascularization, or coronary artery ectasia.

This case had two major criteria and one minor criterion. The angiographic evidence of coronary artery embolism and concomitant embolization at multiple sites are major, and the presence of atrial fibrillation in the patient is a minor criterion. Based on the NCVC criteria, the patient was classified as Definite Coronary Embolism.

In literature, there is no consensus regarding optimal therapeutic algorithms for the management of embolic MI. In the review by Shibata et al., 58% of patients underwent percutaneous coronary intervention (PCI), 97% of these patients had thrombus aspiration as the initial strategy [11]. Conservative management was observed in 42% of the patients with distal occlusion or small vessel involvement. Hernández et al. successfully treated three patients of coronary embolism with transluminal coronary angioplasty and stenting [16]. Pifarre et al. reported the use of catheter embolectomy combined with aortocoronary vein bypass graft in four patients [17].

Frikha et al. reviewed six patients diagnosed with coronary artery embolism [18]. Risk factors for coronary embolism include rheumatic mitral stenosis, mitral valve replacement, and atrial fibrillation. Three patients were managed with aspiration, while thrombolysis was performed in three cases. All patients had a favorable outcome.

Belli et al. recommended a strategy of combining maximal antiplatelet therapy, glycoprotein IIb/IIIa inhibitor infusion, direct aspiration of thrombus along with adjunctive mechanical protection with a balloon and catheter system to protect from distal embolization [19]. Successful aspiration of thrombus was obtained in seven out of eight attempted procedures, with an inability to negotiate the angulated take-off of the LCX artery in one patient. Small distal emboli not amenable to intervention can be managed with anticoagulation.

Rivaroxaban (Xarelto) is an oral once a day factor Xa inhibitor medication for the prevention of stroke and embolism in atrial fibrillation. The ROCKET AF study verified the safety of rivaroxaban as a substitute to warfarin for the prevention of thromboembolism in non-valvular AF [20]. Discontinuation of rivaroxaban has been attributed to a rebound phenomenon with a higher risk of stroke or thromboembolic phenomenon. Initial trial data showed a similar rate of complications between the drugs; however, the higher event rate was seen with rivaroxaban from three to 30 days after study drug discontinuation.

In our patient, the discontinuation of rivaroxaban increased the risk of embolism. The patient was managed with aspiration thrombectomy with percutaneous transluminal coronary angioplasty of the distal LAD, with the resolution of symptoms.

## Conclusions

Coronary embolism is a rare but known cause of acute myocardial infarction with a higher incidence of all-cause death. Abrupt discontinuation of anticoagulation, particularly rivaroxaban, should bring more attention toward clinical suspicion for coronary embolism in atrial fibrillation patients. This case report demonstrates different risk factors related to CAE, with focus on CAE after anticoagulation discontinuation.
